# News and Notes

**Published:** 1902-02

**Authors:** 


### *NEWS AND NOTES.*

---

THE *Atlanta Medical and Surgical Journal* and *Southern Medical Record* having been consolidated into the ATLANTA JOURNAL-RECORD OF MEDICINE, exchanges will please be sent only to the latter. We are getting many duplicates.

---

SIR WILLIAM MACCORMAC, President of the Royal College of Surgeons of England, died suddenly on December 4th, at Bath, aged 65 years.

---

THE controversy over the site for the New York Hospital for Consumptives is ended. Governor Odell has approved the choice of Raybrook in the Adirondacks.

---

DR. WILLIAM K. VAN REYPEN has been renominated by President Roosevelt for another term as Surgeon-general of the U. S. Navy. Dr. van Reypen was entitled to retire on December 18, and it had been popularly supposed that he would be succeeded by Dr. Rixey, who was the medical attendant of the late President McKinley.

---

A QUACK in Königsburg is said to have announced a new cure for consumption. It consists in stopping respiration in the affected lung, so keeping the diseased lung at rest. You go on breathing with the other lung. Very simple. You stop up the nostril on the affected side. Two errors are to be guarded against—stopping the wrong nostril and stopping both.

---

OF 12,500 bottles of quinine tablets (300 tablets to the bottle) recently furnished to the medical department of the U. S. Army in the Philippines, 12,227 bottles were returned to the contractors on account of adulteration. The contractors averred that the fraud was not theirs, but that they had themselves been imposed upon. They replaced the rejected tablets with others of standard purity.

---

THE latest infant prodigy lives in Bridgeville, Del. Professor Wiley, of the Bridgeville Academy, has a son, twenty-one months old, who can spell words with toy blocks. This kid was recently sitting on the floor with a newspaper, apparently reading. When questioned by its father the child showed an acquaintance with the contents of the paper. That boy needs watching. He will presently be smoking cigarettes. There was probably a prodigy in an earlier generation.

---

A MEDICAL "gold brick" recently sold to the daily press attributes to a Pittsburg physician the discovery of a new serum for the cure of tetanus. The doctor is represented as contrasting the ease of application and sure results of his own serum with the use and effects of the old serum to the great disadvantage of the latter. For instance, he says that the ordinary tetanus serum must be poured in by the quart through a trephine opening.

---

PHILADELPHIA has had 127 deaths from smallpox in the year just closed. There were 125 new cases reported during the week ending December 14, when 349 cases were under treatment. One physician has contracted the disease. There are 260 cases of smallpox in the municipal Hospital. The city needs more hospital room for smallpox cases, but is being obstructed at every turn by property owners, who fear that a new hospital will injure their property.

